# It is time to rethink randomized clinical trial approaches

**DOI:** 10.1093/oncolo/oyae172

**Published:** 2024-07-10

**Authors:** David J Stewart

**Affiliations:** Department of Medicine, University of Ottawa, Ottawa, ON K1H 8L6, Canada

## Abstract

This letter to the editor comments on the recently published editorial by Fojo, LaRose, and Bates, and agrees that changes are needed in clinical research approaches.

In their commentary,^1^ Fojo et al make excellent points. We agree there is ample evidence that sotorasib benefits patients with *KRAS G12C* mutations, effective lines of therapy are all important, sotorasib did not need to beat docetaxel, and many trials seriously challenge equipoise. Oncologists *should* do what they deem best for their patients.

It makes little sense for randomized controlled trials (RCTs) to compare drugs with differing mechanisms of action.^2^ Therapies may preferentially benefit different subpopulations. The rational approach is to offer one *then* the other, not one *instead of* the other. One might reasonably assess optimal drug sequence (A->B vs B->A), but it is unreasonable to discard one based on A-vs-B RCTs if each may benefit patients refractory to the other.^2^

In CodeBreak200,^3^ median progression-free survival (PFS) was 1.1 months longer with sotorasib vs docetaxel. However, as frequently happens,^4^ medians were distorted by Kaplan-Meier midpoint deviations. Half-life assessments mitigate this. Curve exponential decay nonlinear regression analysis using published methods^4^ indicated PFS half-lives of 6.1-vs-4.3 months with sotorasib-vs-docetaxel.

Hazard ratios correlate better with half-life ratios than with median ratios.^4^ This was the case here (PFS hazard ratio 0.66, half-life ratio 4.3/6.1 = 0.70, and median ratio 4.5/5.6 = 0.80).

For most therapies, if overall survival (OS) is undistorted by crossover, PFS half-life gains ≥ 1.5 months indicate a 90% probability of OS gains ≥ 2 months.^5^ Crossover ≥ 20% substantially reduces probability that OS differences will achieve significance.^4^ While OS is important, it can be an unreliable RCT endpoint due to crossover^4^ and long post-progression survival.^6^ We agree with Fojo et al,^1^ we must rethink OS as a primary RCT endpoint.^5^

We also agree that further trials should primarily assess who is *most likely* to benefit. This is most efficiently done using response as the outcome measure.^2^ This can subsequently be confirmed with non-randomized PFS/OS assessments. Subsequent assessment of real-world patients managed using different approaches can elucidate whether a factor is prognostic rather than predictive. We do not need RCTs for this.

Fojo et al are correct that targeted therapies can be toxic.^1^ However, the VP4-2023 trial demonstrated that dose reductions reduced sotorasib toxicity much more than efficacy.^7^ Many therapies have relatively flat dose-response curves.

With respect to setting the bar higher,^1^ it depends. Using RCTs to assess efficacy requires high accrual to show small gains.^8^ This slows progress by deviating resources from assessment of other options. Far fewer patients are needed to demonstrate high gains.^2^ A higher bar reduces trial costs.

However, only 20-30 patients are needed in phases I-II trials if single agent response rate is used to assess efficacy. In 25 trials of placebo/best supportive care, median objective response rate was 1%.^2^ The highest response rate was 4%. Hence, if even a minority of patients (≥10%?) experience durable responses, this may indicate a drug worth trying in patients with no good alternatives if toxicity and price are reasonable. RCTs are needed to confirm modest contributions of a drug to a combination,^2^ but are unnecessary with single agents.

Overall, we should rethink RCT approaches in assessing new therapies.^2^

## References

[1] Fojo T , LaRose M, Bates SE. The impact of exuberance on equipoise in oncology clinical trials: sotorasib as archetype. Oncologist. 2024;29(4):275-277. 10.1093/oncolo/oyae042 38498045 PMC10994247

[2] Stewart DJ , Kurzrock R. Fool’s gold, lost treasures, and the randomized clinical trial. BMC Cancer. 2013;13:193. 10.1186/1471-2407-13-193 23587187 PMC3639810

[3] de Langen AJ , Johnson ML, Mazieres J, et al; CodeBreaK 200 Investigators. Sotorasib versus docetaxel for previously treated non-small-cell lung cancer with KRAS(G12C) mutation: a randomised, open-label, phase 3 trial. Lancet. 2023;401(10378):733-746. 10.1016/S0140-6736(23)00221-0 36764316

[4] Stewart DJ , Bosse D, Goss G, et al. A novel, more reliable approach to use of progression-free survival as a predictor of gain in overall survival: The Ottawa PFS Predictive Model. Crit Rev Oncol Hematol. 2020;148:102896. 10.1016/j.critrevonc.2020.102896 32087510

[5] Stewart DJ , Ramsay T, Navani V, et al. Progression-free survival gain: a reliable primary end point for drug registration that can accelerate patient access to urgently needed therapies. J Clin Oncol. 2024;42(8):973-974. 10.1200/JCO.23.02259 38290085

[6] Broglio KR , Berry DA. Detecting an overall survival benefit that is derived from progression-free survival. J Natl Cancer Inst. 2009;101(23):1642-1649. 10.1093/jnci/djp369 19903805 PMC4137232

[7] Hochmair MJ , Vermaelen K, Mountzios G, et al. VP4-2023: Sotorasib 960 mg versus 240 mg in pretreated KRAS G12C advanced NSCLC. ESMO Virtual Plenary Abstracts. Ann Oncol. 2024;35(1):142-144. https://www.annalsofoncology.org/article/S0923-7534(23)04996-7/pdf

[8] Stewart DJ , Kurzrock R. Cancer: the road to Amiens. J Clin Oncol. 2009;27(3):328-333. 10.1200/JCO.2008.18.9621 19064964

